# Systems-Based Practice Core Competency: A Survey of Physicians' Perspectives

**DOI:** 10.7759/cureus.109676

**Published:** 2026-05-26

**Authors:** Ami L DeWaters, Britta L Thompson, Lindsay Mazotti, Erin Miller, Nardine Riegels, Dan Wolpaw, Catherine Coe, Craig Collins, Robert Cooney, Paul Haidet, Grant Harrell, James B Reilly, Nick Sawyer, Catherine Witkop, Linda Montgomery, Terry Wolpaw, Jed Gonzalo

**Affiliations:** 1 Internal Medicine, Penn State College of Medicine, Hershey, USA; 2 Assessment and Evaluation, Penn State College of Medicine, Hershey, USA; 3 Internal Medicine, Sutter Health, Oakland, USA; 4 Biostatistics, RTI Health Solutions, Hershey, USA; 5 Internal Medicine, Kaiser Permanente Oakland Medical Center, Oakland, USA; 6 Family Medicine, University of California San Diego, San Diego, USA; 7 Surgery, Kaiser Permanente West Los Angeles Medical Center, Los Angeles, USA; 8 Emergency Medicine, Geisinger Medical Center, Danville, USA; 9 Medicine, Penn State College of Medicine, Hershey, USA; 10 Family Medicine, University of Florida, Old Town, USA; 11 Internal Medicine, Allegheny Health Network, Pittsburgh, USA; 12 Emergency Medicine, University of California (UC) Davis Health, Sonoma, USA; 13 Obstetrics and Gynecology, Uniformed Services University of the Health Sciences, Besthesda, USA; 14 Family Medicine, University of Colorado Anschutz Medical Campus, Denver, USA; 15 Internal Medicine, Virginia Tech Carilion School of Medicine, Roanoke, USA

**Keywords:** acgme core competencies, assessment in health professions education, clinical features, graduate medical education (gme), survey research

## Abstract

Background: Systems-Based Practice (SBP) has been a core competency in the United States Graduate Medical Education (GME) since 1999, but little is known about physicians' attitudes toward and skills in SBP.

Methods: The aim of this study was to examine resident and GME faculty physicians' perceptions of their engagement, responsibility, and skills in SBP and opportunities to engage in SBP within the clinical workplace. A cross-sectional survey was conducted from May to June 2021 of 6,899 resident and GME faculty physicians across 10 health systems. The survey used Likert-scale questions to assess participants' perceptions across four domains, including engagement in SBP, responsibility for SBP, self-perceived SBP knowledge and skills, and affordances in the clinical environment to learn SBP.

Results: The survey response rate was 21% (n=1,436). Most respondents were engaged in SBP and believed it was their responsibility to engage in SBP. The minority of respondents reported having adequate skills in several core SBP domains, including contributing to system-level improvements (n=576; 40.1%), addressing social determinants of health (n=530; 36.9%), and engaging in population health management (n=348; 24.2%). Organizational affordances had lower average scores.

Conclusions: Resident and GME faculty physicians seem to endorse a high degree of responsibility for and engagement in SBP while identifying substantial self-perceived skill deficits in SBP. Participants reported insufficient affordances in the clinical environment to develop several SBP sub-competencies.

## Introduction

In 1999, the Accreditation Council for Graduate Medical Education (ACGME) implemented Systems-Based Practice (SBP) as one of six core competencies in graduate medical education (GME) [1]. SBP includes critical components of effective healthcare delivery, such as high-value care, interprofessional collaboration, social determinants of health, population health management, patient safety, and quality improvement [2,3]. SBP has been defined as "understanding the interdependencies of a system or series of systems and the changes identified to improve care that can be made and measured in the system" [2]. Despite having been established over 20 years ago, meaningful implementation of SBP into GME programs has not been realized [4,5]. One challenge has been the definition of SBP itself, which has suffered from "construct ambiguity" [6]. Without a clear understanding, SBP has been difficult to teach and assess. Additionally, while there has been increased integration of SBP into undergraduate medical education, it is unclear if those who have received this education have noted improved SBP abilities due to that education [2,6,7].

Another challenge for SBP has been the difficulty of integrating this learning into the clinical environments where SBP is practiced, learned, and taught [2]. Residents are primarily learning in the clinical workplace rather than in didactic classrooms. Therefore, opportunities (or "affordances") for engaging in SBP learning must be readily available so that residents and faculty can practice, learn, and teach SBP [8-10]. Affordances are defined in workplace learning theory as the factors in the workplace that positively enable workers to learn [8]. We sought to understand how faculty and residents perceive SBP affordances, responsibility, engagement, and abilities in the workplace, but limited studies have explored this concept in GME. Given that health systems recognize SBP as a critical component in effective healthcare delivery, it is imperative to take a hard look at how workplace environments are encouraging or discouraging the practice of SBP [11-15]. In this study, we examined resident and faculty physicians' perceptions of their engagement, responsibility, and abilities in SBP and opportunities to engage in SBP within the clinical workplace. The overarching goal of this work was to elucidate how SBP is currently viewed by residents and GME faculty.

## Materials and methods

Design and participants

Study Design, Participants, and Setting

Resident and faculty physicians at 10 sponsoring institutions of US GME programs were invited to participate in a quantitative, cross-sectional survey. The health systems were chosen for a diversity of program characteristics, including US geographic region, setting, type (non-for-profit, military), and academic affiliation (Table 1) to reflect the diversity of all GME programs. Institutions were also selected because they had previously expressed interest to the research team in SBP-related work. Participants were limited to those in the 11 largest (by resident number) ACGME-accredited GME specialty programs: anesthesiology, emergency medicine, family medicine, internal medicine, neurology, obstetrics/gynecology, orthopedic surgery, pediatrics, psychiatry, surgery, and radiology [16]. Since we sought to assess GME workplace environments in a comprehensive manner and we hypothesized that resident and faculty physicians would have different perspectives, we invited both resident physicians and core faculty (as defined by the ACGME) within each program to participate [17].

**Table 1:** Participating institutions and resident and core faculty physicians invited to complete the survey ^a^Residency programs/specialties: 1: anesthesiology; 2: emergency medicine; 3: family medicine; 4: internal medicine; 5: neurology; 6: obstetrics and gynecology; 7: orthopedics surgery; 8: pediatrics; 9: psychiatry; 10: radiology; and 11: surgery

Sponsoring institution	City/state, region, setting, and description	Residency programs included^a ^	Residents invited	Faculty invited
Penn State College of Medicine	Hershey, Pennsylvania; mid-Atlantic region; rural/suburban setting; integrated academic health center with a college of medicine in a public health science university. Family medicine programs located in Hershey, State College, and Reading, PA	1, 2, 3, 4, 5, 6, 7, 8, 9, 10, 11	385	264
Allegheny Health Network	Pittsburgh, Pennsylvania; mid-Atlantic region; urban setting; subsidiary of the integrated healthcare delivery and financing system, Highmark Health. Family medicine and emergency medicine programs in Pittsburgh and Erie, PA	1, 2, 3, 4, 5, 6, 7, 9, 10, 11	370	188
Geisinger Health Network and School of Medicine	Danville, Pennsylvania; mid-Atlantic region; rural/urban/suburban setting; health system with multispecialty training in Danville. Family medicine, internal medicine, and surgery programs in Wilkes-Barre/Scranton, PA, and family medicine in Lewistown, PA	1, 2, 3, 4, 5, 6, 7, 8, 10, 11	314	212
Kaiser Permanente Southern California	Los Angeles, California; far west region; urban setting; integrated health system with graduate medical education programs at several medical centers in southern California. Family medicine programs in San Bernardino County, LAMC, Riverside, San Diego, Woodland Hills, and Orange County, CA, and internal medicine programs in San Bernardino County and LAMC	2, 3, 4, 5, 6, 8, 9, 10, 11	329	415
Kaiser Permanente Northern California	Oakland, California; far west region; urban setting; integrated health system with graduate medical education programs at seven medical centers in northern California. Family medicine programs in Napa/Solano, San Jose, and Santa Rosa; internal medicine programs in Oakland, San Francisco, and Santa Clara; obstetrics and gynecology programs in Oakland, San Francisco, and Santa Clara; pediatrics program in Oakland; and psychiatry programs in Oakland and San Jose, CA	3, 4, 6, 8, 9	286	141
Uniformed Services University/National Capital Consortium	Bethesda, Maryland; mid-Atlantic region; suburban/urban setting; health system with school of medicine (Uniformed Services University) and residency programs in the Washington, D.C. region (Bethesda, MD, and Alexandria, VA)	1, 3, 4, 5, 6, 7, 8, 9, 10, 11	372	172
University of California Davis School of Medicine	Davis, California; far west region; suburban setting; integrated academic health system with school of medicine and residency programs in the Sacramento, CA region	1, 2, 3, 4, 5, 6, 7, 8, 9, 10, 11	495	302
University of Colorado School of Medicine	Aurora, Colorado; Rocky Mountain region; urban setting; integrated academic health system with a school of medicine. Family medicine programs in Aurora (university core and rural tracks) and Denver (Swedish Family Medicine), CO	1, 2, 3, 4, 5, 6, 7, 8, 9, 10, 11	693	381
University of Florida College of Medicine	Gainesville, Florida; southeast region; urban/suburban setting; integrated academic health center with a college of medicine	1, 2, 3, 4, 5, 6, 7, 8, 9, 10, 11	527	273
University of North Carolina School of Medicine	Chapel Hill, North Carolina; southeast region; urban setting; integrated academic health center with the University of North Carolina Hospitals	1, 2, 3, 4, 5, 6, 7, 8, 9, 10, 11	512	258

Survey Instrument Creation and Validity Evidence

To provide content validity evidence for the survey, we created a team of seven experts: three with expertise in survey design and medical education and four with expertise in SBP [18]. We conducted an extensive literature review and identified relevant frameworks to thoroughly define the concept and determine if surveys already existed that could be used or adapted. Our literature review yielded three frameworks, which we used to develop our survey: workplace learning, health systems science, and clinical learning environment (CLE) [8-11,19]. In the context of workplace learning, we specifically focused on the combination of personal engagement and workplace affordances: in order for meaningful learning to occur in the workplace, individuals must be engaged, and the setting must allow for learning [8-10]. The health systems science framework was chosen because it provided a comprehensive framework for SBP. Health systems science includes patient safety, health system improvement, high-value care, social determinants of health, population health management, interprofessional teamwork, health information technology, transitions of care, and advocacy for quality care [11]. Finally, we drew upon the clinical learning environment framework as proposed by Gruppen et al., which categorizes the CLE into four categories: social factors, organizational factors, physical factors, and personal factors [19].

The expert team used these three conceptual frameworks to create four overarching constructs in the survey. The constructs were faculty/resident engagement in SBP, faculty/resident responsibility for SBP, self-perceived knowledge/skills in SBP, and affordances of the clinical environment to learn SBP. The team initially created 50 items. These items were then rated for representativeness, clarity, and relevance by four additional experts who were not part of the survey development team. Based on these reviews, the survey was reduced to 40 items.

To ensure response process validity evidence, we conducted cognitive interviews with 24 resident and faculty physicians, which allowed us to pilot test the survey and explore respondents' interpretation(s) of each question using immediate retrospective probing [20]. These participants were selected from five different specialties and were representative of the overall study sample. Interviews featured verbal probes which asked respondents' interpretations of questions and their confidence in their answers and to restate the question [20]. Based on these interviews, 33 items were kept, and three were modified for increased clarity, resulting in a final survey that included 36 items (see Appendices). All items used a five-point response scale (engagement/responsibility: 1=not at all true and 5=very true; ability: 1=very poor and 5=excellent; and skill/affordances: 1=very poor, 2=poor, 3=acceptable, 4=very good, and 5=excellent). This survey development process was performed in alignment with Messick's framework [21].

To examine the internal structure of the survey (i.e., validity evidence regarding internal structure), we used factor analysis with both orthogonal and oblique rotation to identify latent dimensions [20-23]. We wanted to ensure that the questions under each of the four overarching constructs would be appropriate to group together. We examined the scree plot, eigenvalues ≥1.0, and a minimum of two items with loadings >0.40 on a dimension. To provide evidence of both internal structure validity and internal consistency reliability, Cronbach's alpha was used.

Data collection

Between May 18 and June 30, 2021, we invited participants via email to complete the survey. Each site co-investigator led the outreach to study participants at their institution. The email described the study, explained the voluntary nature of the project, identified that informed consent was implied by survey completion, and included a survey link. Respondents were provided an option to enter a raffle to win one of the 320 $25 gift certificates. Participants received an initial invitation to complete the questionnaire with three additional follow-up reminders.

Statistical analysis

Descriptive statistics included mean scores for each of the constructs. Responses to individual items were combined to calculate a top-box percentage to aid in interpretation; for example, "very good" and "excellent" were combined into one response. To determine non-response bias in our sample, we used wave analysis. Briefly, we multiplied the proportion of non-respondents by the mean response of the first-wave respondents (initial invitation up to the first reminder) minus the mean response of the last-wave respondents (final reminder to the close of the questionnaire) [21-25]. The mean responses of the last-wave respondents are a proxy for non-respondents. Inferential statistics included multivariate analysis of variance (repeated measures MANOVA) of each construct in addition to correlation analysis (Pearson's product-moment correlation). We identified two a priori comparisons for the analysis. First, we compared faculty and resident physician responses to elucidate whether faculty and resident physicians have different engagement in SBP or interpretation of affordances. Second, we compared participants who had received (and not received) education in SBP during medical school to elucidate differences in perceptions regarding the responsibility and engagement in SBP. In addition to statistical significance, we also calculated educational significance. For educational significance, meaning changes that would be noticeable in an educational environment, we used effect size analysis of either eta-squared (η2) or Cohen's d. We considered small effects as 0.20/0.01 (Cohen's d/η2), medium effects as 0.50/0.06 (Cohen's d/η2), and large effects as ≥0.80/0.14 (Cohen's d/η2) [17]. Data were analyzed using IBM SPSS Statistics for Windows, Version 27.0 (IBM Corp., Armonk, New York, United States) [26].

Ethical clearance

The study was approved by the Institutional Review Board of Penn State College of Medicine (approval number: STUDY00017264). Each collaborating institution, when required, received approval from its institutional review board.

## Results

Of the 6,899 resident and faculty physicians invited, 1,436 completed the survey for an overall response rate of 21%. This included 686 faculty physicians (26%) and 750 resident physicians (16%); see Table 2. The minority of respondents reported having adequate skills in several core SBP domains, including contributing to system-level improvements (n=576; 40.1%), addressing social determinants of health (n=530; 36.9%), and engaging in population health management (n=348; 24.2%). The minority of respondents also reported working in an environment where attendings give formal feedback on SBP (n=435; 30.3%), they receive formal didactics on SBP (n=497; 34.6%), and the physical layout of the hospital supported SBP (n=559; 38.9%).

**Table 2:** Characteristics of resident and faculty physicians at 10 US academic health systems

Characteristics	Total, n (%)	Resident physicians, n (%)	Faculty physicians, n (%)
Total respondents	1,436	750	686
Gender			
Male	660 (45.9)	336 (44.8)	324 (47.2)
Female	686 (47.7)	378 (50.4)	308 (44.8)
Non-binary	4 (0.3)	1 (0.1)	3 (0.4)
Prefer not to answer	84 (5.8)	35 (4.7)	51 (7.4)
Ethnicity			
White: non-Hispanic	863 (60)	421 (56.1)	442 (64.3)
Asian/Pacific Islander	250 (17.4)	157 (20.9)	93 (13.5)
Black: non-Hispanic	35 (2.4)	14 (1.9)	21 (3.1)
Hispanic	68 (4.7)	35 (4.7)	33 (4.8)
Native American/Alaskan Native	1 (0.1)	1 (0.1)	-
Other	77 (5.4)	43 (5.7)	-
Prefer not to answer	152 (10.6)	75 (10)	77 (11.2)
Specialty			
Anesthesiology	87 (6.1)	56 (7.5)	31 (4.5)
Emergency medicine	83 (5.8)	42 (5.6)	41 (6)
Family medicine	248 (17.2)	138 (18.4)	110 (16)
Internal medicine	316 (22)	202 (26.9)	114 (16.6)
Neurology	46 (3.2)	15 (2)	31 (4.5)
Obstetrics/gynecology	143 (9.9)	53 (7.1)	90 (13.1)
Orthopedic surgery	50 (3.5)	25 (3.3)	25 (3.6)
Pediatrics	164 (11.4)	80 (10.7)	84 (12.2)
Psychiatry	92 (6.4)	49 (6.5)	43 (6.3)
Radiology	72 (5)	31 (4.1)	41 (6)
Surgery	111 (7.7)	52 (6.9)	59 (8.6)
Other	25 (1.7)	7 (0.9)	18 (2.6)
Affiliation			
Penn State College of Medicine	184 (12.8)	96 (12.8)	88 (12.8)
Allegheny Health Network	133 (9.2)	63 (8.4)	70 (10.2)
Kaiser Permanente: Northern	229 (16.8)	134 (17.9)	95 (13.8)
Kaiser Permanente: Southern	173 (11.2)	69 (9.2)	104 (15.1)
Geisinger Commonwealth	126 (8.8)	48 (6.4)	78 (11.4)
University of Florida	41 (2.9)	17 (2.3)	24 (3.5)
University of North Carolina	104 (7.2)	49 (6.5)	55 (8)
University of California at Davis	115 (8)	75 (10)	40 (5.8)
University of Colorado	186 (12.9)	109 (14.5)	77 (11.2)
Uniformed Services University/National Capital Consortium	146 (10.2)	90 (12)	56 (8.2)
Training year			
PGY1	-	218 (29.1)	-
PGY2	-	214 (28.5)	-
PGY3	-	201 (26.8)	-
PGY4	-	65 (8.7)	-
PGY5	-	18 (2.4)	-
>PGY5	-	8 (1)	-
No answer	-	26 (3.5)	-
Faculty years since completing residency/fellowship			
0-3	-	-	64 (9.7)
4-6	-	-	97 (14.7)
7-9	-	-	95 (14.4)
>10	-	-	200 (30.2)
>20	-	-	94 (14.2)
>30	-	-	111 (16.8)

Non-response bias was calculated using wave analysis for each of the four scales. The non-response bias in mean score differences between the first wave and the last wave was as follows: ability=0.01, engagement=0.02, responsibility=0.05, and affordances=0.14. These differences, given they were on a five-point scale, were considered to be small.

Factor analysis 

Factor loading indicated that five items loaded on engagement, nine items on responsibility, nine items on ability (knowledge/skills), and 11 items on affordances, with a Cronbach's alpha of 0.85, 0.82, 0.85, and 0.93, respectively. See Table 3 for factor analysis.

**Table 3:** Factor analysis using principal component analysis with varimax rotation of Systems-Based Practice items of faculty and resident physicians All items were on the five-point Likert scale. *Even though these two items loaded onto two factors at 0.4 and above, we chose to include them in the factor on which they loaded the highest.

	Affordances	Responsibility	Engagement	Ability
Do you believe…				
It is important for you to be trained in Systems-Based Practice?	-	-	0.797	-
It is necessary to use Systems-Based Practice to deliver optimal patient care?	-	-	0.779	-
You are interested in participating in Systems-Based Practice?	-	-	0.749	-
You have more to learn about Systems-Based Practice?	-	-	0.484	-
All physicians should be competent (i.e., knowledgeable and skilled) in the components of Systems-Based Practice?	-	-	0.773	-
Do you believe you should take personal responsibility for…				
Identifying and reporting patient safety gaps?	-	0.537	-	-
Contributing to system-level improvements that address barriers to optimal patient care?	-	0.620	-	-
Applying principles of high-value care in medical decision-making?	-	0.474	-	-
Addressing social determinants of health (e.g., income, access to care, food insecurity, etc.) that influence a patient's health?	-	0.772	-	-
Improving the health of the population in the community in which you work?	-	0.790	-	-
Encouraging collaboration with interprofessional members of your care team (e.g., nurses, therapists, pharmacists, physicians, etc.)?*	-	0.488	0.448	-
Effectively using health information technology (e.g., electronic health records, registries, telehealth) to deliver optimal patient care?	-	0.482	-	-
Coordinating the transition of care for a patient between providers and healthcare settings (e.g., hospitals, nursing homes, and clinics)?	-	0.604	-	-
Advocating for quality patient care and optimal patient care systems?	-	0.467	-	-
How would you rate your personal ability to…				
Identify and report patient safety gaps?	-	-	-	0.717
Contribute to system-level improvements that address barriers to optimal patient care?	-	-	-	0.711
Apply principles of high-value, cost-conscious care in medical decision-making?	-	-	-	0.651
Address social determinants of health (e.g., income, access to care, food insecurity, etc.) that influence a patient's health?	-	-	-	0.479
Engage in population health management?*	-	0.424	-	0.490
Collaborate with interprofessional members of the care team (e.g., nurses, therapists, pharmacists, physicians, etc.)?	-	-	-	0.627
Effectively use health information technology (e.g., electronic health records, registries, telehealth) to deliver optimal patient care?	-	-	-	0.600
Coordinate the transition of care for a patient between providers and healthcare settings (e.g., hospitals, nursing homes, and clinics)?	-	-	-	0.623
Advocate for quality patient care and optimal patient care systems?	-	-	-	0.708
How would you rate the quality of…				
Attendings' skills in Systems-Based Practice?	0.762	-	-	-
Residents' skills in Systems-Based Practice?	0.693	-	-	-
Attendings' role modeling of Systems-Based Practice?	0.795	-	-	-
The quality of collaboration on Systems-Based Practice initiatives (e.g., patient safety projects, system improvement projects, etc.) between your residency program and the hospital/clinic in which you primarily work?	0.750	-	-	-
The level of support from your residency program to include Systems-Based Practice in clinical care?	0.763	-	-	-
Formal education and training activities residents receive in Systems-Based Practice?	0.747	-	-	-
Feedback attendings give residents in Systems-Based Practice?	0.753	-	-	-
Medical decision-making that you observe regarding high-value care?	0.729	-	-	-
Interprofessional care teamwork, inclusive of nurses, therapists, pharmacists, physicians, etc.?	0.638	-	-	-
Health information technology (e.g., electronic health records, clinical information systems, etc.) used to enhance patient care?	0.625	-	-	-
Transitions of care for a patient between providers and healthcare settings (e.g., hospitals, nursing homes, and clinics)?	0.684	-	-	-
The physical layout of the hospital/clinics in terms of its ability to allow care teams to work well together?	0.618	-	-	-
The physical layout of the hospital/clinics in terms of its ability to allow for effective patient care delivery?	0.627	-	-	-

Engagement, responsibility, ability, and affordances in SBP

Table 4 reports the mean results of engagement, responsibility, ability, and affordances in SBP.

**Table 4:** Mean engagement, responsibility, ability, and affordances scores on a five-point Likert scale regarding Systems-Based Practice among faculty and resident physicians in 10 US academic health systems ^a^Percentage of respondents who rated the item as either a 4 or a 5 on the 1-5 Likert scale, i.e., a "top box" score. Bold was used to highlight items that had a minority of respondents.

Survey item	Resident physicians, mean (SD); %^a^	Faculty physicians, mean (SD); %^a ^	Total, mean (SD); %^a^
Engagement (Do you believe…)			
It is important for you to be trained in Systems-Based Practice?	4.41 (0.71); 90	4.60 (0.69); 93.7	4.50 (0.71); 91.8
It is necessary to use Systems-Based Practice to deliver optimal patient care?	4.34 (0.79); 84.3	4.58 (0.71); 92.6	4.45 (0.76); 89.8
You are interested in participating in Systems-Based Practice?	4.22 (0.80); 83.2	4.48 (0.78); 89.5	4.34 (0.80); 86.3
You have more to learn about Systems-Based Practice?	4.53 (0.69); 93.3	4.38 (0.76); 91.6	4.46 (0.73); 92.5
All physicians should be competent (i.e., knowledgeable and skilled) in the components of Systems-Based Practice?	4.40 (0.71); 90.5	4.57 (0.68); 92	4.48 (0.70); 91.2
Responsibility (Do you believe you should take personal responsibility for…)			
Identifying and reporting patient safety gaps?	4.61 (0.63); 96.3	4.74 (0.56); 97.1	4.67 (0.60); 96.7
Contributing to system-level improvements that address barriers to optimal patient care?	4.40 (0.69); 92	4.61 (0.65); 95.2	4.50 (0.68); 93.5
Applying principles of high-value, cost-conscious care in medical decision-making?	4.58 (0.70); 94.3	4.70 (0.61); 96.4	4.63 (0.66); 95.3
Addressing social determinants of health (e.g., income, access to care, food insecurity, etc.) that influence a patient's health?	4.43 (0.84); 90.3	4.40 (0.89); 87.6	4.42 (0.86); 89
Improving the health of the population in the community in which you work?	4.03 (0.97); 77.2	4.06 (1.0); 74.7	4.04 (0.98); 76
Encouraging collaboration with interprofessional members of your care team (e.g., nurses, therapists, pharmacists, physicians, etc.)?	4.70 (0.61); 96.4	4.81 (0.48); 98	4.75 (0.55); 97.1
Effectively using health information technology (e.g., electronic health records, registries, telehealth) to deliver optimal patient care?	4.66 (0.63); 96.8	4.75 (0.55); 97.4	4.70 (0.6); 97.1
Coordinating the transition of care for a patient between providers and healthcare settings (e.g., hospitals, nursing homes, and clinics)?	4.31 (0.96); 86.3	4.52 (0.85); 91	4.41 (0.92); 88.5
Advocating for quality patient care and optimal patient care systems?	4.80 (0.50); 98.4	4.84 (0.47); 8.5	4.82(0.49); 98.5
Ability (How would you rate your personal ability to…)			
Identify and report patient safety gaps?	3.34 (0.76); 36.7	3.85 (0.85); 66.5	3.59 (0.84); 51.2
Contribute to system-level improvements that address barriers to optimal patient care?	3.07 (0.83); 26	3.59 (0.94); 55.2	3.32 (0.92); 40.1
Apply principles of high-value, cost-conscious care in medical decision-making?	3.47 (0.81); 47.3	3.88 (0.87); 67.9	3.67 (0.86); 57.4
Address social determinants of health (e.g., income, access to care, food insecurity, etc.) that influence a patient's health?	3.19 (0.90); 35.8	3.21 (1.03); 38.4	3.20 (0.96); 36.9
Engage in population health management?	2.81 (0.89); 17.8	3.06 (1.03); 31.5	2.93 (0.96); 24.2
Collaborate with interprofessional members of the care team (e.g., nurses, therapists, pharmacists, physicians, etc.)?	4.13 (0.74); 80.1%	4.37 (0.75); 87.9	4.24 (0.76); 83.8
Effectively use health information technology (e.g., electronic health records, registries, telehealth) to deliver optimal patient care?	3.83 (0.80); 67.2	4.08 (0.85); 76.9	3.95 (0.84); 72.1
Coordinate the transition of care for a patient between providers and healthcare settings (e.g., hospitals, nursing homes, and clinics)?	3.42 (0.85); 44.4	3.69 (0.96); 60.6	3.55 (0.91); 52.2
Advocate for quality patient care and optimal patient care systems?	3.80 (0.84); 67.1	4.15 (0.83): 79.7	3.96 (0.86); 73.4
Affordances (How would you rate the quality of…)			
Attendings' skills in Systems-Based Practice?	3.72 (0.76); 62.7	3.66 (0.78); 60.4	3.69 (0.77); 61.6
Residents' skills in Systems-Based Practice?	3.42 (0.74); 43.4	3.38 (0.81); 42.9	3.40 (0.77); 43.2
Attendings' role modeling of Systems-Based Practice?	3.57 (0.82); 53.8	3.51 (0.82); 50.7	3.54 (0.82); 52.3
The quality of collaboration on Systems-Based Practice initiatives (e.g., patient safety projects, system improvement projects, etc.) between your residency program and the hospital/clinic in which you primarily work?	3.53 (0.82); 50.5	3.52 (0.92); 51.1	3.53 (0.87); 50.8
The level of support from your residency program to include Systems-Based Practice in clinical care?	3.60 (0.91); 55.6	3.70 (0.89); 60.6	3.65 (0.90); 58
Formal education and training activities residents receive in Systems-Based Practice?	3.18 (0.90); 34.6	3.25 (0.87); 36.8	3.22 (0.88); 35.7
Feedback attendings give residents in Systems-Based Practice?	3.04 (0.93); 30.3	3.08 (0.91); 29.3	3.06 (0.92); 29.8
Medical decision-making that you observe regarding high-value care?	3.58 (0.84); 52.8	3.53 (0.83); 51.8	3.56 (0.83); 52.3
Interprofessional care teamwork, inclusive of nurses, therapists, pharmacists, physicians, etc.?	3.94 (0.84); 71.7	3.96 (0.83); 73	3.95 (0.83); 72.3
Health information technology (e.g., electronic health records, clinical information systems, etc.) used to enhance patient care?	3.76 (0.96); 63	3.81 (0.95); 65.4	3.78 (0.96); 64.1
Transitions of care for a patient between providers and healthcare settings (e.g., hospitals, nursing homes, and clinics)?	3.53 (0.84); 50.8	3.58 (0.89); 55.5	3.55 (0.86); 53
The physical layout of the hospital/clinics in terms of its ability to allow care teams to work well together?	3.29 (0.95); 38.9	3.17 (1.02); 35.8	3.23 (0.99); 37.4
The physical layout of the hospital/clinics in terms of its ability to allow for effective patient care delivery?	3.42 (0.91); 44.5	3.31 (1.01); 41	3.36 (0.96); 42.9

The overwhelming majority of respondents reported it was important for physicians to be engaged in SBP (86-92%) and that they had a responsibility to the domains of SBP (76-99%). Participants' perceived ability in SBP was lower, ranging from 24% to 84%. In addition, participants' perception of affordances in the CLE was also low, ranging from 30% to 72%. We also noted differences in the mean score ratings across each of the four domain areas (Table 3). These ratings across domain areas by each participant were both statistically and educationally significant. Specifically, we noted higher ratings for the domains of engagement (4.45; SD: 0.58) and responsibility (4.55; SD: 0.47) and lower ratings for ability (3.60; SD: 0.59) and affordances (3.50; SD: 0.64).

Simple correlation analysis (Pearson's product-moment correlation) indicated that responsibility and engagement were moderately positively correlated (r=0.478) and ability and affordances were positively and moderately to strongly correlated (r=0.548).

Differences between and within participants 

Table 5 compares the responses in the four domains between faculty and resident physicians and participants who did or did not receive SBP education in medical school and ratings within each participant group in the four domains.

**Table 5:** Differences between faculty and residents' and participants' receipt of Systems-Based Practice education in medical school with the assessment of self-perceived engagement, responsibility, ability, and affordances regarding Systems-Based Practice in 10 US academic health systems Bold was used to highlight items with lower means.

Domains	Role				Education in Systems-Based Practice in medical school				Overall		
	Faculty, mean (SD; N=686)	Residents, mean (SD; N=750)	p	Cohen's d	Yes, mean (SD; N=557)	No, mean (SD; N=880)	p	Cohen's d	Overall, mean (SD; N=1437)	p	η2
Engagement	4.52 (0.56)	4.38 (0.59)	<0.001	0.25	4.51 (0.52)	4.48 (0.57)	0.48	0.04	4.45 (0.58)	<0.001	0.58
Affordances	3.50 (0.65)	3.50 (0.64)	0.82	0.01	3.64 (0.63)	3.42 (0.64)	<0.001	0.35	3.50 (0.64)		
Responsibility	4.60 (0.44)	4.51 (0.48)	<0.001	0.21	4.58 (0.45)	4.57 (0.43)	0.78	0.02	4.55 (0.47)		
Ability	3.77 (0.60)	3.46 (0.54)	<0.001	0.54	3.64 (0.58)	3.64 (0.61)	0.91	0.01	3.60 (0.59)		

Faculty and resident responses were significantly different, with faculty rating all four domains higher: engagement (faculty=4.52 vs. residents=4.38; p<0.001), responsibility (faculty=4.60 vs. residents=4.51; p<0.001), and ability (faculty=3.77 vs. residents=3.46; p<0.001). Only ratings for ability were both statistically and educationally significant. Most respondents reported not having a formal education in SBP. Participants who had received SBP education during medical school had slightly higher ratings compared with those who had not. However, the difference in affordances between the two groups was the only domain that was both statistically and educationally significant (SBP education=3.64 vs. no SBP education=3.42; p<0.001).

Specialty-specific results 

We identified statistically and educationally significant differences in specialty areas using MANOVA with Roy's largest root (F(3, 1434); p<0.001; η2=0.075). In the domain of responsibility, anesthesiology and emergency medicine were significantly lower than pediatrics and family medicine. In the domain of ability, anesthesiology, emergency medicine, radiology, surgery, internal medicine, and psychiatry were significantly lower than obstetrics/gynecology, pediatrics, and family medicine. For engagement, obstetrics/gynecology and family medicine were significantly higher rated than all other specialties. Finally, in terms of affordances, family medicine was significantly higher than surgery, anesthesiology, and emergency medicine (Table 6).

**Table 6:** Comparing engagement, responsibility, affordances, and ability across 11 specialty areas ^a^Means are taken from Likert-scale responses that ranged from 1 to 5.

	N	Responsibility		Ability		Affordances		Engagement	
		Mean^a ^	Standard deviation	Mean^a ^	Standard deviation	Mean^a ^	Standard deviation	Mean^a^	Standard deviation
Family medicine	248	4.70	0.4	4.56	0.5	3.76	0.7	3.69	0.6
Pediatrics	164	4.64	0.3	4.50	0.5	3.51	0.6	3.67	0.6
Obstetrics/gynecology	143	4.62	0.4	4.55	0.5	3.61	0.6	3.69	0.5
Psychiatry	91	4.59	0.4	4.49	0.6	3.57	0.7	3.57	0.6
Internal medicine	316	4.57	0.4	4.48	0.6	3.55	0.7	3.59	0.6
Surgery	111	4.51	0.6	4.28	0.7	3.27	0.7	3.59	0.6
Neurology	46	4.49	0.5	4.38	0.6	3.47	0.6	3.55	0.6
Orthopedics	50	4.47	0.5	4.15	0.8	3.50	0.6	3.55	0.5
Radiology	72	4.40	0.6	4.43	0.7	3.64	0.6	3.58	0.7
Emergency medicine	83	4.34	0.5	4.32	0.6	3.36	0.6	3.48	0.6
Anesthesia	87	4.29	0.7	4.30	0.6	3.31	0.6	3.41	0.6

## Discussion

The results of this cross-sectional survey study suggest that although current-day resident and faculty physicians believe they should take responsibility for and have engagement in SBP, they report less ability in SBP domains. In addition, their assessment of SBP affordances is significantly less positive than their perception of their own engagement in SBP. This gap between responsibility and ability, as well as the gap between engagement and affordances, was particularly pronounced in domains related to social determinants of health and population health management. This is perhaps not surprising given that these concepts were not emphasized in initial descriptions of SBP [1]. There have been calls recently to establish a new competency to address population health and social determinants more directly [27]. However, the overall gaps in the operationalization of SBP suggest that the establishment of a new competency may not fully address the underlying driving factors of these disconnects. Notably, affordances were the lowest rated of the four domains. It was notable that all four domains (ability, engagement, responsibility, and affordances) were positively correlated with each other. This may provide validity to a recent conceptual model for SBP affordances, which outlined the need to consider data from across health system institutions as educators implement and assess SBP [28]. For example, residents may struggle to develop skills in quality improvement if they work in a health system that lacks a robust quality department.

It is notable that previous SBP education during medical school did not generate a discernible effect on self-perceived SBP ability. This mirrors findings from another study that suggested primarily teaching SBP in a classroom setting is suboptimal and, instead, SBP should be integrated into the teaching and daily routine of the clinical learning environment, which again highlights the importance of affordances [28]. However, those who did receive education in SBP in medical school rated their current affordances in GME more highly than those who had not. This raises the question of whether being exposed to SBP in medical school primed applicants to search and apply to GME programs that demonstrated evidence of SBP principles in their clinical learning environments. In addition, there is the possibility that residents who have received more exposure to SBP are more aware of SBP initiatives occurring in their workplace environments. Whether or not increased awareness of SBP could eventually lead to increased SBP skill remains unclear and may be an area of future research.

With few exceptions, family medicine, pediatrics, and obstetrics/gynecology faculty and residents rated each of the four domains higher than other specialties. Considering these are primary care specialties, this finding raises the possibility that there are structural or process differences in these specialties' clinical environments, ACGME specialty requirements, or GME programs that have generated different affordances that may lead to a higher uptake of SBP. If such specialty differences exist, there may be an opportunity to translate those best practices into other practice contexts and specialties. These findings are a prime area for additional future research to better understand the link between affordances and ability.

While these results provide an important first step toward integrating SBP in clinical practice, several limitations must be considered. First, institutions that participated in the study had previously expressed interest in SBP research, potentially introducing selection bias. These institutions may be more supportive of SBP, leading to more favorable results. Additionally, while our 21% response rate aligns with other national resident surveys, non-responder bias may limit the generalizability of our findings [17]. Although wave analysis suggested non-responders' results would have been similar to our responders', it remains possible that those who participated had a greater interest in SBP, positively skewing the results [29]. As a result, physicians may feel less engaged and responsible for SBP than our data suggest, though the gap between responsibility and ability would likely persist as non-responders would also likely rate their skills less positively as well.

Another limitation is the timing of data collection, which occurred during the COVID-19 pandemic. The disruptions of the pandemic may have influenced responses, though the broader challenges of SBP integration likely remain relevant. Finally, while physician self-perceptions provide valuable insight into the SBP implementation challenge, they are not sufficient on their own [30]. These findings are not reflective of observed SBP skills, and future research should examine physicians' actual SBP-related skills and assess patient-level outcomes to gain a more comprehensive understanding of SBP competence.

## Conclusions

SBP is widely recognized as a core physician competency, and both resident and faculty physicians appear to endorse this perspective. This study implies that physicians feel engaged and feel responsible for SBP. However, those same physicians did not seem to think highly of their skills in SBP and affordances for SBP in GME clinical learning environments. This could represent an opportunity to focus future SBP education.

## Appendices

Survey instrument

Survey Description

The following survey relates to your experiences in your current residency program.  On this survey, you will see the term "Systems-Based Practice". While many programs may define Systems-Based Practice in their own unique way, for the purpose of THIS survey, please consider "Systems-Based Practice" to mean the overall ability of trainees and faculty to work effectively across varied healthcare delivery settings and systems, coordinate patient care within the healthcare system, address the social determinants of health (e.g., food insecurity, housing insecurity, access to care, insurance, etc.) for individual patients and populations of patients, be aware of the economic costs of healthcare, and incorporate these into decision-making for individual patients, advocate for and participate in quality improvement of healthcare settings and systems, work effectively with other professions (e.g., nurses, pharmacists, other specialties, etc.) to provide safe and effective patient care, as part of routine practice, identify healthcare system issues that create medical errors, and participate in implementing potential systems solutions.

NOTE: If you are a faculty member, please consider your experiences in the residency program in which you CURRENTLY WORK as an attending physician, NOT the program(s) where you trained.

Engagement  

Scale: 1=not at all true; 2=somewhat untrue; 3=uncertain; 4=somewhat true; 5=very true 

Do you believe:  

1. It is important for you to be trained in Systems-Based Practice?    

2. It is necessary to use Systems-Based Practice to deliver optimal patient care?    

3. You are interested in participating in Systems-Based Practice?    

4. You have more to learn about how to apply Systems-Based Practice to your clinical care?

5. All physicians should be competent (i.e., knowledgeable and skilled) in the components of Systems-Based Practice?  

Responsibility

Scale: 1=not at all true; 2=somewhat untrue; 3=uncertain; 4=somewhat true; 5=very true

Do you believe you should take personal responsibility for:

1. Identifying and reporting patient safety gaps?  

2. Contributing to system-level improvements that address barriers to optimal patient care?  

3. Applying principles of high-value care in medical decision-making?  

4. Addressing social determinants of health (e.g., income, access to care, food insecurity, etc.) that influence a patient's health?  

5. Engaging in population health management?  

6. Encouraging collaboration with interprofessional members of your care team (e.g., nurses, therapists, pharmacists, physicians, etc.)?  

7. Effectively using health information technology (e.g., electronic health records, registries, telehealth) to deliver optimal patient care? 

8. Coordinating the transition of care for a patient between providers and healthcare settings (e.g., hospitals, nursing homes, and clinics)?   

9. Advocating for optimal patient care? 

Skills 

Scale: 1=very poor; 2=poor; 3=acceptable; 4=very good; 5=excellent

How would you rate your personal ability to:  

1. Identify and report patient safety gaps?  

2. Contribute to system-level improvements that address barriers to optimal patient care?  

3. Apply principles of high-value care in medical decision-making?  

4. Address social determinants of health (e.g., income, access to care, food insecurity, etc.) that influence a patient's health?  

5. Engage in population health management?  

6. Collaborate with interprofessional members of the care team (e.g., nurses, therapists, pharmacists, physicians, etc.)?  

7. Effectively use health information technology (e.g., electronic health records, registries, telehealth) to deliver optimal patient care?

8. Coordinate the transition of care for a patient between providers and healthcare settings (e.g., hospitals, nursing homes, and clinics)?   

9. Advocate for optimal patient care? 

Sociocultural Affordances for Systems-Based Practice in Clinical Learning Environments

Scale: 1=very poor; 2=poor; 3=acceptable; 4=very good; 5=excellent

Please answer the following items about the residency or fellowship program in which you work.  

How would you rate:

1. Attending physicians' skills in Systems-Based Practice?  

2. Resident physicians' skills in Systems-Based Practice?  

3. Attending physicians' role modeling of Systems-Based Practice?    

4. The quality of collaboration on Systems-Based Practice initiatives (e.g., patient safety projects, system improvement projects, etc.) between your residency or fellowship program and the hospital/clinic in which you primarily work?  

5. The level of support from your residency or fellowship program to include Systems-Based Practice in clinical care?   

Organizational Affordances for Systems-Based Practice in Clinical Learning Environments

Scale: 1=very poor; 2=poor; 3=acceptable; 4=very good; 5=excellent

Please answer the following items about the residency or fellowship program in which you work.  

How would you rate the quality of:  

1. Formal education and training activities residents receive in Systems-Based Practice?   

2. Feedback attending physicians give residents in Systems-Based Practice?    

3. Medical decision-making that you observe regarding high-value, cost-conscious care?  

4. Interprofessional care teamwork, inclusive of nurses, therapists, pharmacists, physicians, etc.?  

5. Health information technology (e.g., electronic health records, clinical information systems, etc.) used to enhance patient care?  

6. Transitions of care for a patient between providers and healthcare settings (e.g., hospitals, nursing homes, and clinics)?   

Please answer the following items about the teams in the clinical units (e.g., outpatient clinic, inpatient wards, operating room, etc.) on which you work, inclusive of the nurses, case managers, clinic managers, staff, administrators, and other physicians, that are part of those teams. 

How would you rate the team's ability to:  

1. Proactively identify and address patient safety gaps?  

2. Eliminate barriers to optimal patient care?  

3. Address social determinants of health (e.g., income, access to care, food insecurity, etc.) that influence a patient's health?  

4. Improve the health of the population in the community in which you work?  

5. Advocate for quality patient care and optimal patient care systems?   

Physical Environment Affordances for Systems-Based Practice in Clinical Learning Environments

Scale: 1=very poor; 2=poor; 3=acceptable; 4=very good; 5=excellent

Please answer the following items in regard to the primary hospital or clinic in which you work most frequently.  

How would you rate:  

1. The physical layout of the hospital/clinics in terms of its ability to allow care teams to work well together?  

2. The physical layout of the hospital/clinics in terms of its ability to allow for effective patient care delivery?

Demographics

1. Please indicate your school affiliation:  

a. Penn State College of Medicine

b. Allegheny Health Network

c. Kaiser Permanente Northern California

d. Kaiser Permanente Southern California

d. Geisinger Health Network

e. Penn State College of Medicine

f. Uniformed Services University/National Capital Consortium

g. University of California at Davis  

h. University of Colorado

i. University of Florida 

j. University of North Carolina

2. Please indicate your gender:  

a. Male

b. Female

c. Non-binary

d. Prefer not to disclose 

3. How do you identify your race?  

a. White

b. Asian or Asian American 

c. Black or African American

d. Native Hawaiian or Pacific Islander

e. American Indian or Alaskan Native

f. Something else

f. Prefer not to disclose 

4. How do you identify your ethnicity?

a. Hispanic or Latino

b. Non-Hispanic or Latino

c. Prefer not to disclose

5. Please identify your specialty:  

a. Anesthesiology

b. Emergency medicine

c. Family medicine

d. Internal medicine

e. Neurology

f. Obstetrics/gynecology

g. Orthopedic surgery

h. Pediatrics

i. Psychiatry

j. Radiology

k. Surgery

l. Other: Please specify____________

6. Please indicate your current role:  

a. Resident physician

b. Faculty/practicing physician

7. Please indicate your role as a faculty member:

a. Program director

b. Assistant or associate program director

c. Core faculty

8. Please indicate your training year:  

a. PGY1

b. PGY2

c. PGY3

d. PGY4

e. PGY5

f. PGY6

g. PGY7

h. PGY8

i. PGY9

j. PGY10

Please indicate your faculty year (number of years since completing residency/fellowship):

___________

10. I received education and/or training specifically related to Systems-Based Practice in medical school.   

a. Yes

b. No

c. Not sure

11. I received education and/or training specifically related to Systems-Based Practice in my residency training program.      

a. Yes

b. No

c. Not sure

12. If you would like to have your name entered into a raffle to win a $25 gift certificate, please click this link to submit your name.

LINK: _______________________________
